# Etomoxir Sodium Salt Promotes Imidazole Ketone Erastin-Induced Myeloid-Derived Suppressor Cell Ferroptosis and Enhances Cancer Therapy

**DOI:** 10.3390/biology13110949

**Published:** 2024-11-19

**Authors:** Nada Mohamady Farouk Abdalsalam, Zihao Liang, Hafiza Kashaf Tariq, Abdulrahman Ibrahim, Rong Li, Xiaochun Wan, Dehong Yan

**Affiliations:** 1 Guangdong Immune Cell Therapy Engineering and Technology Research Center, Center for Protein and Cell-Based Drugs, Institute of Biomedicine and Biotechnology, Shenzhen Institute of Advanced Technology, Chinese Academy of Sciences, Shenzhen 518055, China; nadamohamady@siat.ac.cn (N.M.F.A.); zh.liang@siat.ac.cn (Z.L.); hafizakashaftariq@siat.ac.cn (H.K.T.); abdulrahman@siat.ac.cn (A.I.);; 2 University of Chinese Academy of Sciences, Beijing 100864, China

**Keywords:** etomoxir sodium salt, imidazole ketone erastin, myeloid-derived suppressor cell, ferroptosis, cancer therapy

## Abstract

Although ferroptosis inducers trigger ferroptotic tumor cells and immune cells in the tumor microenvironment (TME), imidazole ketone erastin (IKE)’s induction of ferroptosis shows no effect on tumor growth in immunocompetent tumor-bearing mice due to the presence of myeloid-derived suppressor cells (MDSCs). Treatment of the carnitine palmitoyltransferase 1a (CPT1A)-specific inhibitor decreases the immunosuppressive function of MDSCs and enhances ferroptotic inducer-initiated tumor cell ferroptosis. However, whether blocking CPT1A could enhance IKE-induced MDSC ferroptosis and thereby inhibit tumor growth is still unclear. Here, we report that a CPT1A-specific inhibitor, etomoxir sodium salt (Eto), and IKE combined treatment increased MDSC ferroptosis. Interestingly, the combination treatment of Eto and IKE blocked MDSCs’ immunosuppressive function and accumulation by downregulating the expression of SLC7A11, GPX4, and ARG1 while promoting T-cell proliferation and infiltration into tumor tissues to enhance cancer therapy. These data provide a rationale for the combination therapy of a specific CPT1A inhibitor, Eto, with IKE in clinical settings.

## Simple Summary

Targeting ferroptosis provides new therapeutic opportunities to treat refractory cancers, but myeloid-derived suppressor cells (MDSCs) in the tumor microenvironment (TME) hinder the curative effect of ferroptosis-induced cancer therapy. This study demonstrated that blocking CPT1A by etomoxir sodium salt (Eto) augments imidazole ketone erastin (IKE)-induced MDSC actual ferroptotic death and blocks MDSC immunosuppressive function and accumulation by breaking the SLC7A11 and GPX4 ferroptosis defense systems and downregulating the expression of ARG1, which impair tumor growth via promoting T-cell proliferation and infiltration into tumor tissues.

## 1. Introduction

Targeting ferroptosis provides new therapeutic opportunities to treat refractory cancers that show powerlessness to conventional therapies [1,2,3]. Ferroptosis is an iron-dependent form of regulated cell death driven by an overload of ROS-triggered lipid peroxidation in cellular membranes, which are characterized by constricted mitochondria and reduced mitochondrial biogenesis [4,5,6,7]. Breaking the ferroptotic defense systems, mainly including the solute carrier family 7-member 11 (SLC7A11)–glutathione peroxidase 4 (GPX4) system, is crucial for developing ferroptosis [1,2,8,9,10]. SLC7A11 imports extracellular cystine into cells, and then cystine is converted into cysteine in the cytosol. Subsequently, cysteine (the rate-limiting precursor), glycine, and glutamate synthesize reduced glutathione (GSH), which is used by GPX4 as the cofactor to reduce lipid hydroperoxides to lipid alcohols, thereby suppressing ferroptosis [3,4,5,8,9,11,12]. However, ferroptosis inducers can inhibit SLC7A11-mediated cystine uptake, restrict intracellular cysteine and GSH levels, and decrease GPX4 levels, thus leading to lethal accumulation of lipid peroxidation in cellular membranes and subsequent membrane rupture to develop ferroptosis execution [3,13,14]. In cancer cells, to meet the increased energetic and biosynthetic needs for supporting their rapid proliferation, ferroptosis inducers induce metabolic reprogramming with their unique features, which include the enrichment of polyunsaturated fatty acid (PUFA)-containing phospholipids (PUFA-PLs) and the overload of iron and imbalanced ferroptosis defense systems, thereby creating a targetable vulnerability to ferroptosis in drug-resistant difficult-to-treat cancer cells, such as breast cancer cells, melanoma cells, lung cancer cells, and colon cancer cells [2,3,15,16,17,18,19]. Thus, ferroptosis inducers have great potential in combination with conventional therapies, including chemotherapy and targeted therapies.

In addition to inducing tumor cell ferroptosis, ferroptosis inducers evoke ferroptotic immune cells in the tumor microenvironment (TME) [20,21,22,23]. Ferroptosis can boost antitumor immunity by blocking the accumulation of myeloid-derived suppressor cells (MDSCs), which are a heterogeneous population of immature myeloid cells and are defined as CD45^+^CD11b^+^Gr-1^+^ cells in mouse tumor tissues [24,25,26,27]. They mainly encourage a pro-tumor environment by exerting potent immunosuppressive effects, in which MDSCs can induce T-cell suppression by depleting the extracellular availability of L-arginine via the arginase 1 (ARG-1)-dependent metabolic pathway and dysregulate TCR signaling via reactive oxygen species (ROS)-induced oxidative stress [28,29,30]. In fact, the induction of MDSC ferroptosis via activating the p53-Hmox1 signaling pathway suppresses MDSC aggregation to increase T-cell infiltration and, thus, enhances CD8^+^ T-cell-mediated tumor suppression [31]. Although both the ferroptosis inducers erastin and imidazole ketone erastin (IKE) can trigger MDSC ferroptosis [24,25,26,32], erastin is widely used in cell culture studies, and IKE is more commonly used for in vivo research because IKE has substantially improved potency, metabolic stability, and water solubility compared with erastin [2,3,9,14,16]. Indeed, IKE provokes diffuse large B-cell lymphoma, pancreatic cancer cell, and breast cancer cell ferroptosis and restrains in vivo tumor growth in immunodeficient mice [1,2,3,12,14,16,17]. But recent studies revealed that IKE’s induction of ferroptosis did not inhibit tumor growth in immunocompetent tumor-bearing mice, in which ferroptosis caused polymorphonuclear (PMN)-MDSCs to release more peroxidized lipids, which decreased T-cell survival and impaired T-cell function during the early stage of ferroptosis [20,24,25,26]. These studies demonstrated that the early stage of ferroptosis could promote MDSCs’ immunosuppressive function, but the late stage of ferroptosis may decrease the accumulation and immunosuppression of MDSCs.

Lipid metabolism provides a primary source of energy and the immunosuppressive function of MDSCs in the TME [28,33,34]. Long-chain fatty acids are transported from the cytosol to the mitochondria via the carnitine palmitoyltransferase (CPT) system, in which CPT1A catalyzes the rate-limiting step of fatty acid oxidation (FAO) for ATP requirements in MDSCs [35,36,37,38,39,40]. Targeting the inhibition of CPT1A by a specific inhibitor, etomoxir, decreases fatty acid uptake, ATP production, arginase I (ARG1) expression, and the immunosuppressive activity of MDSCs [41,42]. Interestingly, a recent report showed that the blockade of CPT1A by etomoxir promoted ferroptosis inducer erastin-induced lung cancer cell actual ferroptotic death via the GPX4 pathway [43]. However, whether blocking CPT1A could enhance ferroptosis inducer IKE-initiated MDSC actual ferroptotic death and thereby inhibit tumor growth is still unclear. In this study, we provide a rationale for the development of the combination therapy of a specific CPT1A inhibitor, etomoxir sodium salt (Eto), with IKE in clinical settings.

## 2. Materials and Methods

### 2.1. Mice

The mice were maintained under a 12 h light–12 h dark cycle in the Animal Facilities of the Shenzhen Institute of Advanced Technology (SIAT), the Chinese Academy of Sciences (CAS). C57BL/6 mice (6–8-week-old females) were obtained from Zhuhai Bestest Biotechnology Co., Ltd. (Zhuhai, China).

### 2.2. Cell Lines

The CAS cell bank provided the mouse tumor B16F10 (melanoma) cells, LLC (Lewis lung carcinoma) cells, and MC38 cells (colorectal carcinoma) cells, which are refractory and vulnerable to ferroptosis [3,44,45,46], which were then cultivated in DMEM supplemented with 10% FBS and 1% penicillin–streptomycin (PS) at 37 °C and under 5% CO_2_. The medium was changed every 2–3 days, and cell passage was performed once the cells grew to about 90% confluence. In total, 1 × 10^6^ LLC, 1 × 10^6^ B16F10, and 2 × 10^6^ MC38 cells were harvested for injection into C57BL/6 mice to build the tumor-bearing mice.

### 2.3. Solutions/Chemicals

DMEM, RPMI 1640, FBS, PBS, and PS were acquired from VivaCell (Shanghai, China). IKE and Eto were purchased from TargetMol (Boston, MA, USA). Propidium iodide solution (PI) and carboxyfuorescein diacetate succinimidyl ester (CFSE) were purchased from BioLegend (San Diego, CA, USA) and Invitrogen (Carlsbad, CA, USA), respectively.

### 2.4. Reagents

The Zombie NIR Fixable Viability Kit was purchased from BioLegend (San Diego, CA, USA). The following fluorescein-conjugated anti-mouse antibodies were purchased from BioLegend: CD11b, Ly-6G/Ly-6C (Gr-1), CD45, CD3, CD19, NK1.1, CD4, and CD8a. The following immunoblotting primary antibodies—GPX4, SLC7A11, and CPT1A—were acquired from Abcam (Cambridge, MA, USA), vinculin from Santa Cruz (Santa Cruz, CA, USA), and ARG-1 from R&D Systems (Minneapolis, MN, USA).

### 2.5. In Vitro MDSC Generation

The MDSCs were sorted by biotin anti-mouse Gr-1 antibodies and mojosort streptavidin nanobeads (BioLegend) from the bone marrows or spleens of MC38 tumor-bearing mice. The purification of the CD11b^+^Gr-1^+^ MDSCs by >90% was confirmed by flow cytometry. Then, the bone marrow (BM)-derived MDSCs were treated in RPMI 1640 supplemented with 10% FBS and 1% PS at 37 °C and under 5% CO_2_ with 40 μM of IKE or 40 μM of Eto, or IKE plus Eto, while DMSO was used as the control, for 24 h.

### 2.6. Flow Cytometry Analysis for Tumor Leukocytes

The LLC tumor tissue from C57BL/6 mice without any ulceration was carefully removed under sterile conditions after sacrifice. The cells were bathed in 70% ethanol for 30 s and digested with collagenase I, II, and IV and hyaluronidase (Sigma, St. Louis, MO, USA) for 1 h at 37 °C [47]. Tumor leukocyte suspensions were collected, washed, and suspended in PBS containing 2% FBS prior to labeling with the indicated antibodies. Cell viability was assessed using the Zombie NIR Fixable Viability Kit. Flow cytometry data were acquired by collecting 1,000,000 live cells using a Cytek^®^ Aurora flow cytometer and analyzed with the SpectroFlo software (https://cytekbio.com/pages/spectro-flo, accessed on 14 November 2024).

### 2.7. Determining Ferroptosis Accordance

The percentages of PI^−^ cell viability, the production of reactive oxygen species (ROS), mitochondrial superoxide anions, fatty acid uptake, lipid droplet formation, mitochondrial iron (Fe^2+^), lipid peroxidation, and the mitochondrial mass in MDSCs were measured by PI staining with 5 μM of chloromethyl-2, 7-dichlorofluorescein diacetate (CM-H_2_DCFDA, Invitrogen, Carlsbad, CA, USA), 5 μM of Mitosox red (Invitrogen, Carlsbad, CA, USA), 10 ug/mL of BODIPY FLC16 (Invitrogen, Carlsbad, CA, USA), 500 ug/mL of BODIPY 493/503 (Invitrogen, Carlsbad, CA, USA), 5 μM of Mito-FerroGreen (Dojindo, Kumamoto, Japan), 500 ug/mL of BODIPY 581/591 C11 (Invitrogen, Carlsbad, CA, USA) and 50 nM of Mitotracker green (Invitrogen, Carlsbad, CA, USA), respectively [48].

### 2.8. Quantitative Real-Time PCR (qRT-PCR)

RNAiso Plus (Takara, Tokyo, Japan) was used to extract the total RNA of MDSCs, and they were quantified using a NanoDrop 2000c Spectrophotometer (Thermo Scientific, Waltham, MA, USA). cDNA was synthesized from the total RNA using PrimeScript™ RT master mix (Takara, Tokyo, Japan). The qRT-PCR was performed as previously described in [48]. The relative gene expression was calculated after correction for β-actin expression using the 2^−ΔΔCt^ method. All primers were synthesized by Genewiz (Suzhou, China)and are shown in Supplementary Table S1.

### 2.9. Immunoblotting

The MDSCs were lysed to extract proteins using RIPA buffer (Beyotime, Shanghai, China) containing phosphatase and protease inhibitors (Roche, Basel, Switzerland). Immunoblotting was conducted with the Bio-Rad system as previously described in [49]. The primary antibodies were overnight incubated at 4 °C. Membrane-bound proteins were detected using an enhanced chemiluminescence substrate (Merck Millipore, Billerica, MA, USA) with an Amersham Imager 600 (GE Healthcare, Little Chalfont, Buckinghamshire, UK). Protein expression levels were detected by gray value analysis using the ImageJ software (version 1.48).

### 2.10. MDSC Inhibition of T-Cell Proliferation Assay

CFSE-labeled CD3^+^ T cells purified from the spleens of naive C57BL/6 mice by the MojoSort™ Mouse CD3 T Cell Isolation Kit (BioLegend, San Diego, CA, USA) activated with 1 μg/mL of plate-bound anti-mouse CD3 (17A2, BioLegend, San Diego, CA, USA) and anti-mouse CD28 (37.51, BioLegend, San Diego, CA, USA) were co-cultured with MDSCs in a 2:1 ratio. The T-cell proliferation was measured by CFSE dilution after 72 h.

### 2.11. In Vivo Experiments

C57BL/6 mice were injected subcutaneously (s.c.) with 1 × 10^6^ LLC (n = 4 mice/group), 1 × 10^6^ B16F10 (n = 3 mice/group), or 2 × 10^6^ MC38 cells (n = 4 mice/group) and treated daily intraperitoneally (i.p.) with 10 mg/kg of IKE, 15 mg/kg of Eto, or IKE plus Eto, while PBS was used as the control group, from day 3 to day 16 for MC38-bearing mice, from day 0 to day 15 for B16F10-bearing mice, and from day 0 to day 21 for LLC-bearing mice after tumor injection. The tumor volume was measured from day 0 to the end, and the tumor weight was measured on the last day.

### 2.12. Statistical Analysis

Each experiment was conducted at least three times unless otherwise indicated. Data analysis was performed by one-way or two-way ANOVA with Tukey’s post-test using GraphPad Prism version 6.0 (Graphpad Software, La Jolla, CA, USA). All data are shown as means ± the standard error of the means (SEMs), and significance was set at *p* < 0.05. In the figures, the asterisks are used as follows: *, *p* ≤ 0.05; **, *p* ≤ 0.01; ***, *p* ≤ 0.001; and ****, *p* ≤ 0.0001; ns, no significant difference.

## 3. Results

### 3.1. IKE and Eto Combined Treatment Increases Actual Ferroptotic Death of MDSCs In Vitro

**Figure 1:**
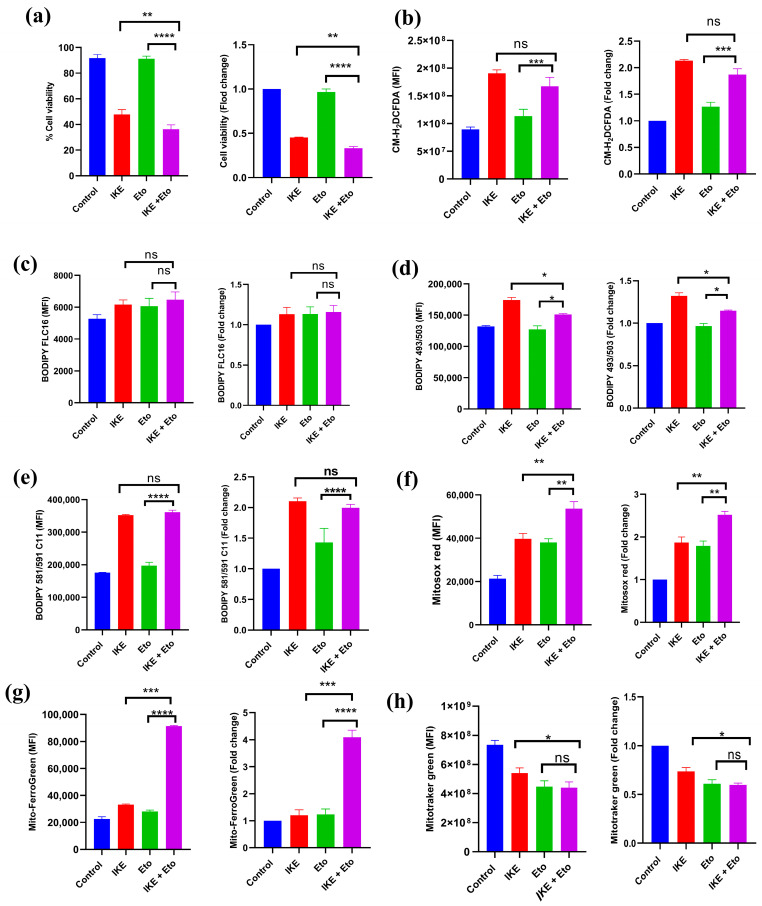
IKE and Eto combined treatment increased MDSCs’ actual ferroptotic death in vitro. Flow cytometry detected the percentages of PI^−^ cell viability and fold change of indicated treatment relative to control group (**a**), mean fluorescence intensity (MFI) of ROS production and fold change of indicated treatment relative to control group (**b**), MFI of fatty acid uptake and fold change of indicated treatment relative to control group (**c**), MFI of lipid droplets and fold change of indicated treatment relative to control group (**d**), MFI of lipid peroxidation and fold change of indicated treatment relative to control group (**e**), MFI of mitochondrial superoxide anions and fold change of indicated treatment relative to control group (**f**), MFI of mitochondrial Fe^2+^ and fold change of indicated treatment relative to control group (**g**), and MFI of mitochondrial mass and fold change of indicated treatment relative to control group (**h**) in in vitro BM-MDSCs. Results from three independent experiments (**a**–**h**). Statistical analysis was performed using one-way ANOVA (**a**–**h**). Data are expressed as means ± SEM (n = 3). * *p* < 0.05, ** *p* < 0.01, *** *p* < 0.001, and **** *p* < 0.0001; ns, no significant difference.

To investigate whether blocking CPT1A could enhance IKE-induced MDSC actual ferroptotic death, in vitro BM-MDSCs, which were isolated from bone marrows of MC38 tumor-bearing mice and directly treated with IKE, Eto, or IKE plus Eto for 24 h, were detected by flow cytometry. Compared with the IKE-treated group, the IKE and Eto combined treatment significantly decreased BM-MDSCs’ viability (Figure 1a and Figure S1a). In addition, when comparing the fold change of the viability of the indicated treatment relative to the control group, we also found that the fold change of the viability of the IKE and Eto combined treatment was lower than that of the IKE-treated group (Figure 1a). Ferroptosis is characterized by upregulating the levels of ROS, fatty acid uptake, lipid peroxidation, mitochondrial superoxide anions, and mitochondrial Fe^2+^ but downregulating the levels of lipid droplets and the mitochondrial mass of cells [3,4,5,6,7]. We detected these factors in in vitro BM-MDSCs by flow cytometry, and we found that compared with the IKE-treated group, the IKE and Eto combined treatment significantly decreased the levels of lipid droplets (Figure 1d and Figure S1d) and mitochondrial mass (Figure 1h and Figure S1g) but did not change the production of ROS (Figure 1b and Figure S1b), the levels of fatty acid uptake (Figure 1c and Figure S1c), and lipid peroxidation (Figure 1e and Figure S1e) while increasing the levels of mitochondrial superoxide anions (Figure 1f and Figure S1f) and mitochondrial Fe^2+^ (Figure 1g and Figure S1h) in BM-MDSCs. Furthermore, when comparing the fold change of the mean fluorescence intensity (MFI) of these factors of the indicated treatment relative to the control group, we also found that compared with the IKE-treated group, the IKE and Eto combined treatment significantly decreased the fold change of the MFI of lipid droplets (Figure 1d) and mitochondrial mass (Figure 1h) but did not change the fold change of the MFI of ROS (Figure 1b), fatty acid uptake (Figure 1c), and lipid peroxidation (Figure 1e) while increasing the fold change of the MFI of mitochondrial superoxide anions (Figure 1f and Figure S1f) and mitochondrial Fe^2+^ (Figure 1g and Figure S1h) in BM-MDSCs. These observations suggest that the specific CPT1A inhibitor Eto enhances IKE-induced BM-MDSC actual ferroptotic death by upregulating mitochondrial superoxide anions and mitochondrial Fe^2+^ levels but downregulating the levels of lipid droplets and mitochondrial mass in vitro.

### 3.2. IKE and Eto Combined Therapy Blocks Accumulation of MDSCs via Increasing Actual Ferroptotic Death of MDSCs In Vivo

**Figure 2:**
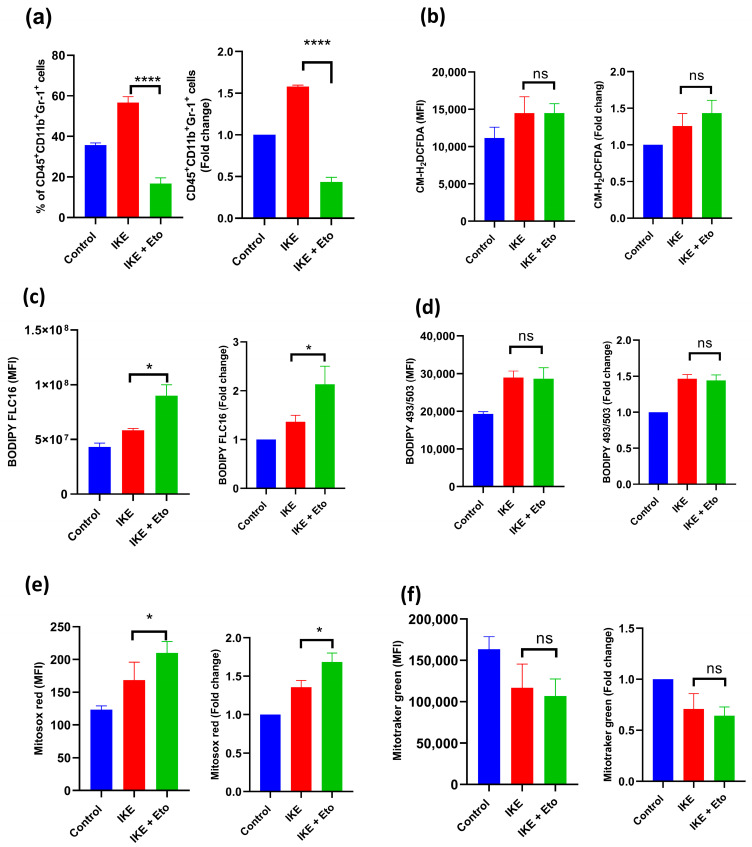
IKE and Eto combined treatment blocked the accumulation of MDSCs in vivo. Flow cytometry detected the percentages of MDSCs (CD45^+^CD11b^+^Gr-1^+^) and fold change of indicated treatment relative to control group (**a**), MFI of ROS and fold change of indicated treatment relative to control group (**b**), MFI of fatty acid uptake and fold change of indicated treatment relative to control group (**c**), MFI of lipid droplets and fold change of indicated treatment relative to control group (**d**), MFI of mitochondrial superoxide anions and fold change of indicated treatment relative to control group (**e**), and MFI of mitochondrial mass and fold change of indicated treatment relative to control group (**f**) in spleen MDSCs of MC38-bearing mice on day 16. Results from three independent experiments (**a**–**f**). Statistical analysis was performed using one-way ANOVA (**a**–**f**). Data are expressed as means ± SEM (n = 3). * *p* < 0.05 and **** *p* < 0.0001; ns, no significant difference.

After 16 days of treatment with 10 mg/kg of IKE, 15 mg/kg of Eto, or both, spleen MDSCs isolated from MC38 tumor-bearing mice were identified by flow cytometry to further examine whether inhibiting CPT1A may improve IKE-induced MDSC actual ferroptotic death in vivo. The percentage of spleen MDSCs (CD45^+^CD11b^+^Gr-1^+^ cells) was significantly lower in the IKE and Eto combined treatment group than in the IKE-treated group (Figure 2a). In addition, when comparing the fold change of the percentage of the indicated treatment relative to the control group, we also found that the fold change of the percentage of the IKE and Eto combined treatment was lower than that of the IKE-treated group (Figure 2a). However, the production of ROS (Figure 2b), the level of lipid droplets (Figure 2d), and mitochondrial mass (Figure 2f) in MDSCs remained unchanged, while the levels of fatty acid uptake (Figure 2c) and mitochondrial superoxide anions (Figure 2e) in MDSCs increased. Furthermore, when comparing the fold change of the MFI of these factors of the indicated treatment relative to the control group, we also found that compared with the IKE-treated group, the IKE and Eto combined treatment significantly increased the fold change of the levels of fatty acid uptake (Figure 2c) and mitochondrial superoxide anions (Figure 2e) but did not affect the production of ROS (Figure 2b), the level of lipid droplets (Figure 2d), or mitochondrial mass (Figure 2f) in MDSCs. These in vitro and in vivo results demonstrate that the combination of IKE and Eto produces more mitochondrial superoxide anions, leading to increased MDSC actual ferroptotic death, thus blocking MDSC accumulation in spleen tissue.

### 3.3. IKE and Eto Combined Treatment Attenuates MDSCs’ Immunosuppressive Function but Strengthens T-Cell Proliferation

**Figure 3:**
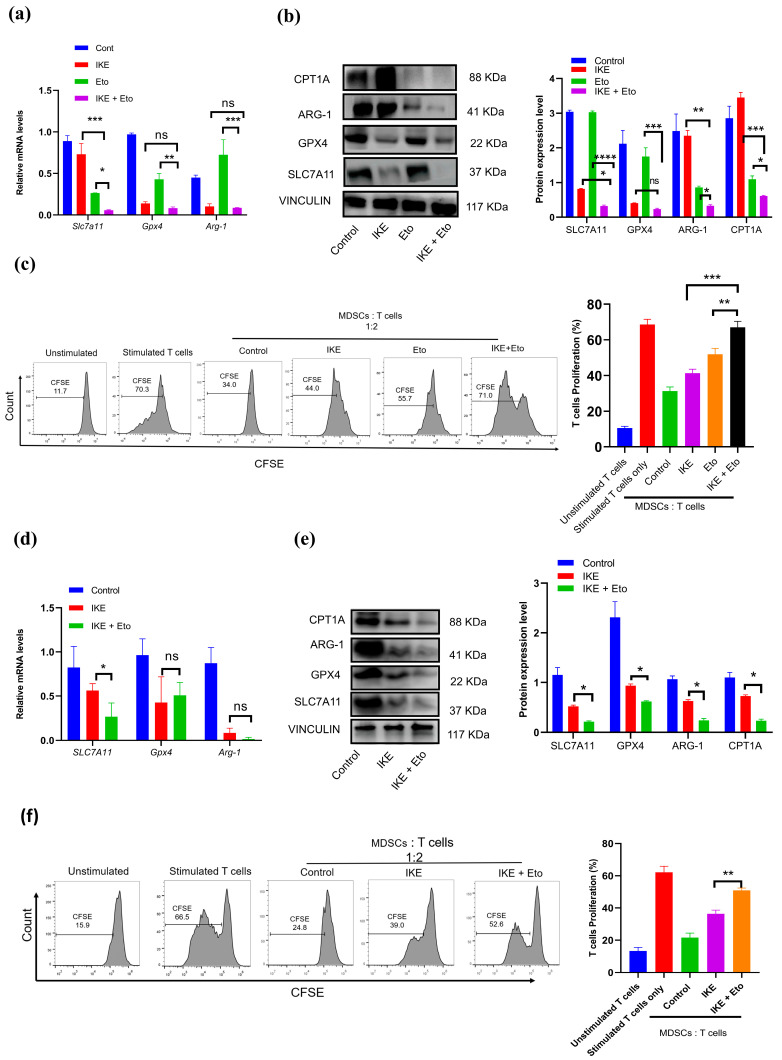
IKE and Eto combined treatment blocked MDSCs’ immunosuppressive function by decreasing anti-ferroptotic and ARG1 proteins, thus promoting T-cell proliferation. The expressions of Slc7a11, Gpx4, and Arg-1 mRNA and proteins in in vitro BM-MDSCs (**a**,**b**) and in vivo spleen MDSCs (**d**,**e**) were detected by RT-qPCR or Western blotting, and protein expression levels were conducted by gray value analysis using ImageJ software (**d**,**e**). Percentages of T-cell proliferation in co-cultures of CFSE-labeled CD3^+^ T cells and in vitro BM-MDSCs (**c**) and in vivo spleen MDSCs (**f**) in a 2:1 ratio were analyzed by flow cytometry. Results from three independent experiments (**a**–**f**). Statistical analysis was performed using one-way ANOVA (**a**–**f**). Data are expressed as means ± SEM (n = 3). * *p* < 0.05, ** *p* < 0.01, *** *p* < 0.001, and **** *p* < 0.0001; ns, no significant difference.

To find out whether the main ferroptosis defense systems contributed to MDSCs’ actual ferroptotic death, we used RT-qPCR and immunoblotting to detect Slc7a11 and Gpx4 mRNA levels and protein expression in in vitro BM-MDSCs. Our results show that the combination treatment of IKE and Eto almost totally inhibited the mRNA and protein expression of SLC7A11 and the GPX4 protein level compared with the IKE-treated group (Figure 3a,b). Previous studies have proven that the specific CPT1A inhibitor etomoxir can decrease ARG1 protein expression in MDSCs and thereby block MDSCs’ immunosuppressive function. We also confirmed that the IKE and Eto combined treatment dramatically reduced the CPT1A and ARG1 protein levels in MDSCs relative to those in IKE-treated MDSCs (Figure 3a,b). Surprisingly, the combined IKE and Eto treatment almost entirely relieved MDSC-mediated T-cell proliferation inhibition (Figure 3c and Figure S2).

Like in the in vitro BM-MDSCs, the combination therapy also greatly reduced the expressions of the Slc7a11 mRNA, SLC7A11, and GPX4 proteins in in vivo spleen MDSCs when compared with those in the IKE-treated group (Figure 3d,e). More importantly, the reduction in the CPT1A and ARG1 proteins in spleen MDSCs led to significantly reactivated T-cell proliferation (Figure 3f). These results suggest that the combined IKE and Eto therapy can not only break the SLC7A11 and GPX4 ferroptosis defense systems to drive MDSCs’ actual ferroptotic death but also attenuate MDSCs’ immunosuppressive function while strengthening T-cell proliferation.

### 3.4. IKE and Eto Combination Therapy Inhibits Tumor Growth by Decreasing Accumulation of MDSCs but Increasing Infiltration of T Cells into Tumor Tissues

**Figure 4:**
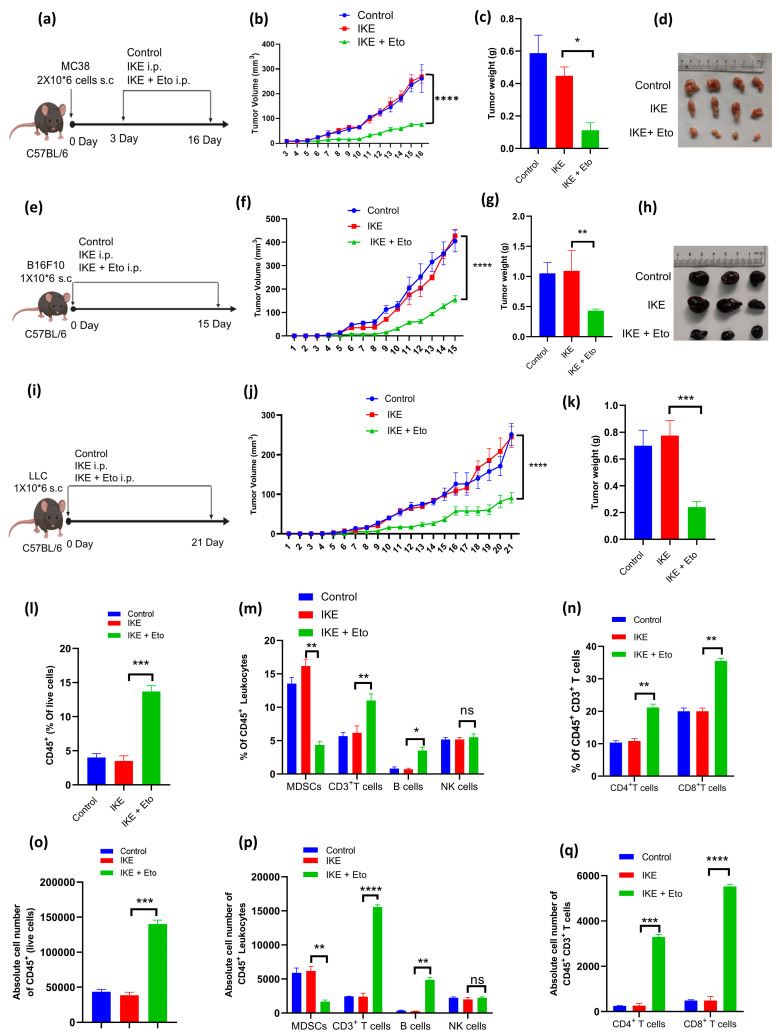
IKE and Eto combination therapy inhibited tumor growth by decreasing accumulation of MDSCs but increasing infiltration of T cells into tumor tissues. MC38 tumor model (n = 4 mice/group): experimental design (**a**), tumor growth curve (**b**), tumor weight (**c**), and image (**d**) on day 16 after tumor injection. B16F10 tumor model (n = 3 mice/group): experimental design (**e**), tumor growth curve (**f**), tumor weight (**g**), and image (**h**) on day 15 after tumor injection. LLC tumor model (n = 4 mice/group): experimental design (**i**), tumor growth curve (**j**), and tumor weight (**k**). The proportions of tumor immune cells in the LLC tumor model on day 21 after tumor injection: (**l**) CD45^+^ leukocytes (CD45^+^ZombieNIR^−^Gated in total single cells); (**m**) MDSCs (CD45^+^CD11b^+^Gr-1^+^), CD3^+^ T cells (CD45^+^CD3^+^), B cells (CD45^+^CD3^−^CD19^+^NK1.1^−^), and NK cells (CD45^+^CD3^−^NK1.1^+^CD19^−^); (**n**) CD4^+^ T cells (CD45^+^CD3^+^CD4^+^CD8^−^) and CD8^+^ T cells (CD45^+^CD3^+^CD4^−^CD8^+^) were detected by flow cytometry and absolute cell number of CD45^+^ leukocytes (**o**); MDSCs, CD3^+^ T cells, B cells, and NK cells (**p**); CD4^+^ T cells and CD8^+^ T cells (**q**) were analyzed by SpectroFlo software. Results from three independent experiments (**b**,**c**, **f**,**g**, **j**,**k** and **l**–**q**). Statistical analysis was performed using one-way ANOVA (**b**,**c**, **f**,**g**, **j**,**k** and **l**–**q**). Data are expressed as means ± SEM (n = 3 or 4). * *p* < 0.05, ** *p* < 0.01, *** *p* < 0.001, and **** *p* < 0.0001; ns, no significant difference.

To investigate the possible synergistic effect of IKE with Eto in suppressing tumor growth, we compared the tumor growth kinetics of C57BL/6 mice s.c. MC38, B16F10, or LLC cells injected with IKE alone or the combined treatment of IKE and Eto. IKE or Eto was administered daily, while the drugs were given starting from day 3 post-tumor inoculation into the mice in the MC38 tumor model (Figure 4a), and the drugs were given starting from day 0 post-tumor inoculation into the mice in the B16F10 and LLC tumor models (Figure 4e,i). Compared with the control-treated mice, no difference in MC38, B16F10, or LLC tumor growth was seen in the IKE alone-treated mice (Figure 4b,f,j), while a significant reduction in tumor growth was observed in all of the above three tumor-bearing mice treated with the IKE and Eto combined therapy (Figure 4b,f,j). More importantly, the combination therapy of IKE and Eto obviously delayed the growth of MC38, B16F10, or LLC tumors when compared with IKE alone-treated tumors (Figure 4b,f,j). Similarly, on day 16, a significant decrease in the MC38 tumor weights in the combination treatment compared with the only IKE-treated group was recorded (Figure 4c). Interestingly, a marked decrease in the B16F10 and LLC tumor weights in the IKE and Eto combination therapy was also noted when compared with the tumor weights in the IKE monotherapy (Figure 4g,k). Moreover, the MC38 and B16F10 tumor sizes in the combined IKE and Eto therapy were smaller than those in the IKE monotherapy (Figure 4d,h).

We next asked whether the combined therapy of IKE and Eto affected the accumulation of MDSCs in tumor tissue, thus contributing to inhibiting tumor growth. By flow cytometry, we detected the proportions of tumor and spleen immune cells in the LLC tumor model on day 21 after tumor injection. We found that the percentages of MDSCs in the tumor and spleen tissues in the IKE and Eto combined treatment were dramatically reduced when compared with those in the IKE monotherapy (Figure 4m, Figures S3a and S4a). However, the percentages of CD45^+^ leukocytes (Figure 4l and Figure S3b), CD3^+^ T cells, B cells (Figure 4m and Figure S3b), CD4^+^ T cells, and CD8^+^ T cells (Figure 4n and Figure S3b) were remarkably increased in the tumors of the combined treatment mice compared with those of the IKE monotherapy mice. In addition, the percentages of NK cells in tumors (Figure 4m and Figure S3b) and CD3^+^ T cells, B cells, NK cells (Figure S4a), CD4^+^ T cells, and CD8^+^ T cells (Figure S4b) in spleen tissues were not different between the IKE and Eto combination therapy and IKE single therapy. Furthermore, we analyzed the absolute cell number of tumor immune cells. We also noticed that the absolute cell number of tumor MDSCs in the IKE and Eto combined treatment was dramatically reduced when compared with that in the IKE monotherapy (Figure 4p). However, the absolute cell numbers of CD45^+^ leukocytes (Figure 4o), CD3^+^ T cells, B cells (Figure 4p), CD4^+^ T cells, and CD8^+^ T cells (Figure 4q) were remarkably increased in the tumors of the combined treatment mice compared with those of the IKE monotherapy mice. However, the absolute cell number of NK cells in tumors (Figure 4p) was not different between the IKE and Eto combination therapy and the IKE single therapy. These results indicate that the IKE and Eto combination therapy inhibited tumor growth by decreasing the accumulation of MDSCs but increasing the infiltration of T cells into tumor tissues.

## 4. Discussion

This study demonstrated that blocking CPT1A by Eto augments IKE-induced MDSC actual ferroptotic death and blocks MDSCs’ immunosuppressive function and accumulation by breaking the SLC7A11 and GPX4 ferroptosis defense systems and downregulating the expression of ARG1, which impair tumor growth via promoting T-cell proliferation and infiltration into tumor tissues.

The functional states of MDSCs in cancer are associated with the different stages of ferroptosis [22,25,26]. In the early stage of ferroptosis, MDSCs under ferroptotic response undergo an active dying state, which is characterized by the uptake and accumulation of PUFA-PLs and their metabolites, such as prostaglandin E2 (PGE2), from the TME [22,26,50]. Acyl-CoA synthetase long-chain family member 4 (ACSL4) and fatty acid transport protein 2 (FATP2) drive PUFA-PLs’ peroxidation to generate ferroptosis signals by cytomembrane-anchored NADPH oxidase 2 (NOX2) [15,18,19,25,33]. The ferroptotic response triggers lipid peroxidation and the amount of ROS released in MDSCs, in which ROS, along with PGE2, contribute to the immunosuppressive activity against antitumor T cells [22,25,26,33,34]. However, the ferroptotic response and ferroptotic cell death are separated in time [22,26,50]. In the late stage of the ferroptotic process, MDSCs eventually die from ferroptosis, which is actual ferroptotic death [22,25,26,50]. In this situation, a significant reduction in MDSCs in the TME can be noted [22,24]. In our study, the combined IKE and Eto treatment overloaded mitochondrial superoxide anions and mitochondrial Fe^2+^ and shattered the SLC7A11 and GPX4 ferroptosis defense systems, thus leading to MDSCs’ actual ferroptotic death and attenuating the accumulation and immunosuppressive function of MDSCs in the TME (Figure 1a,f,g, Figure 2a,e and Figure 3).

Targeting the inhibition of CPT1A provides a trajectory to enhance ferroptosis inducer-based cancer therapy [35,36,39,43]. Cancer cells upregulate the metabolic rate for their rapid proliferation, which creates a PUFA-rich and hypoxic TME, where immune cells are forced to develop pro-ferroptotic stress and generate lipid peroxidation products, thus inducing their greater vulnerability to ferroptotic death than cancer cells [3,16,36,44,46]. This mainly causes antitumor T-cell ferroptotic death and dysfunction because T cells with hypoxia-inducible downregulation of GPX4 accumulate lipid peroxides in cell membranes when entering tumor tissues and subsequently undergo ferroptosis [20,21,22,23]. However, MDSCs under pro-ferroptotic stress upregulate lipid metabolism and transfer PUFAs from the cytosol to the mitochondria via the CPT1A system to spark mitochondrial FAO for ATP requirements [22,24,26,34,41]. MDSCs that obtain sufficient energy will also activate their immunosuppressive function by increasing ARG1 protein expression [28,29,34,41]. In our study, targeting the blockade of CPT1A by Eto dramatically reduced the SLC7A11 and ARG1 gene and protein expression in MDSCs in vitro and in vivo in the tumor microenvironment, leading to not only enhancing IKE-induced MDSC actual ferroptotic death and blocking MDSCs’ immunosuppressive function but also strengthening T-cell proliferation (Figure 3) and infiltration into tumor tissues, thus, ultimately, promoting IKE-triggered tumor growth inhibition (Figure 4).

## 5. Conclusions

This study highlights the crucial role of CPT1A-mediated MDSC ferroptosis resistance through SLC7A11 metabolic reprogramming and MDSCs’ immunosuppressive function via the ARG1 signaling pathway in persisting tumor immune evasion. However, we acknowledge several limitations of this study. There is still a limited understanding of the mechanism of how Eto further lowers IKE-induced SLC7A11 downregulation and which key signaling pathway contributes to this process. Furthermore, whether targeting CPT1A in MDSCs can promote IKE-induced cancer therapy in the clinical setting should be explored in the future. Finally, additional studies are warranted to elucidate the influence of Eto and IKE combination therapeutic treatment on enhancing the efficacy of anti-PD1/PD-L1 cancer immunotherapy.

In summary, these findings offer new insights for the development of a potential therapeutic strategy aimed at disrupting IKE-induced ferroptosis resistance in MDSCs to inhibit tumor progression, with promising clinical implications. Targeting CPT1A represents a previously overlooked approach, synergistic with IKE-induced MDSC actual ferroptotic death in melanoma, lung carcinoma, and colorectal carcinoma.

## Data Availability

The original contributions presented in this study are included in this article. Further inquiries can be directed to the corresponding authors.

## Supplementary Materials

The following supporting information can be downloaded at https://www.mdpi.com/article/10.3390/biology13110949/s1. Figure S1: Representative flow cytometry plots illustrating the gating strategy of cell viability (PI^−^FSC-A^−^), mean fluorescence intensity (MFI) of ROS production, fatty acid uptake, lipid droplets, lipid peroxidation, mitochondrial superoxide anions, mitochondrial mass, and mitochondrial Fe^2+^ in BM-derived CD11b^+^Gr-1^+^ MDSCs treated with IKE or Eto, or both, in vitro for 24 h in Figure 1. Figure S2: Representative flow cytometry plots illustrating the gating strategy of T-cell proliferation in co-cultures of CFSE-labeled CD3^+^ T cells activated with plate-bound anti-mouse CD3 and anti-mouse CD28 and CD11b^+^Gr-1^+^ MDSCs in a 2:1 ratio in Figure 3. Figure S3: Representative flow cytometry plots illustrating the gating strategy of tumor leukocytes in LLC tumor-bearing mice: CD45^+^ leukocytes (CD45^+^ZombieNIR^−^ Gated in total single cells), MDSCs (CD45^+^CD11b^+^Gr-1^+^), CD3^+^ T cells (CD45^+^CD3^+^), B cells (CD45^+^CD3^−^CD19^+^NK1.1^−^), NK cells (CD45^+^CD3^−^NK1.1^+^CD19^−^), CD4^+^ T cells (CD45^+^CD3^+^CD4^+^CD8^−^), and CD8^+^ T cells (CD45^+^CD3^+^CD4^−^CD8^+^) in Figure 4. Figure S4: IKE and Eto combined treatment slowed tumor growth in LLC-bearing mice related to Figure 4. Table S1: Primer sequences used for analyzing transcript levels of genes in in vitro and in vivo CD11b^+^Gr-1^+^ MDSCs in Figure 3.
