# Supplementary material for: Etomoxir Sodium Salt Promotes Imidazole Ketone Erastin-Induced Myeloid-Derived Suppressor Cell Ferroptosis and Enhances Cancer Therapy
Source: Biology (Basel). 2024 Nov 19;13(11):949. doi: 10.3390/biology13110949 (PMC11592117; doi:10.3390/biology13110949)
Supplement: Supplementary file 1 [file biology-13-00949-s001.zip › biology-3271270-supplementary.pdf]

Supplementary data

Supplementary Table S1: Primer sequences used for analyzing the transcript levels of genes in in vitro and in vivo CD11b^{+}Gr-1^{+} MDSCs.

Given names	Primer Sequences
β-Actin MOUSE Forward	CGATATCGCTGCGCTGGTC
β-Actin MOUSE Reverse	AGGTGTGGTGCCAGATCTTC
Slc7a11 MOUSE Forward	ATGGTCAGAAAGCCAGTTGTG
Slc7a11 MOUSE Reverse	GGACAGGGCTCCAAAAAGTG
Gpx4 MOUSE Forward	GCCAAAGTCCTAGGAAACGC
Gpx4 MOUSE Reverse	CCGGGTTGAAAGGTTCAGGA
Gch1 MOUSE Forward	AGCGCCTCACCAAACAGATT
Gch1 MOUSE Revers	GCCAAAGTCCTAGGAAACGC
Arg1 MOUSE Forward	GTGAAGAACCCACGGTCTGT
Arg1 MOUSE Reverse	AGAAAGGACACAGGTTGCCC

Supplementary figures

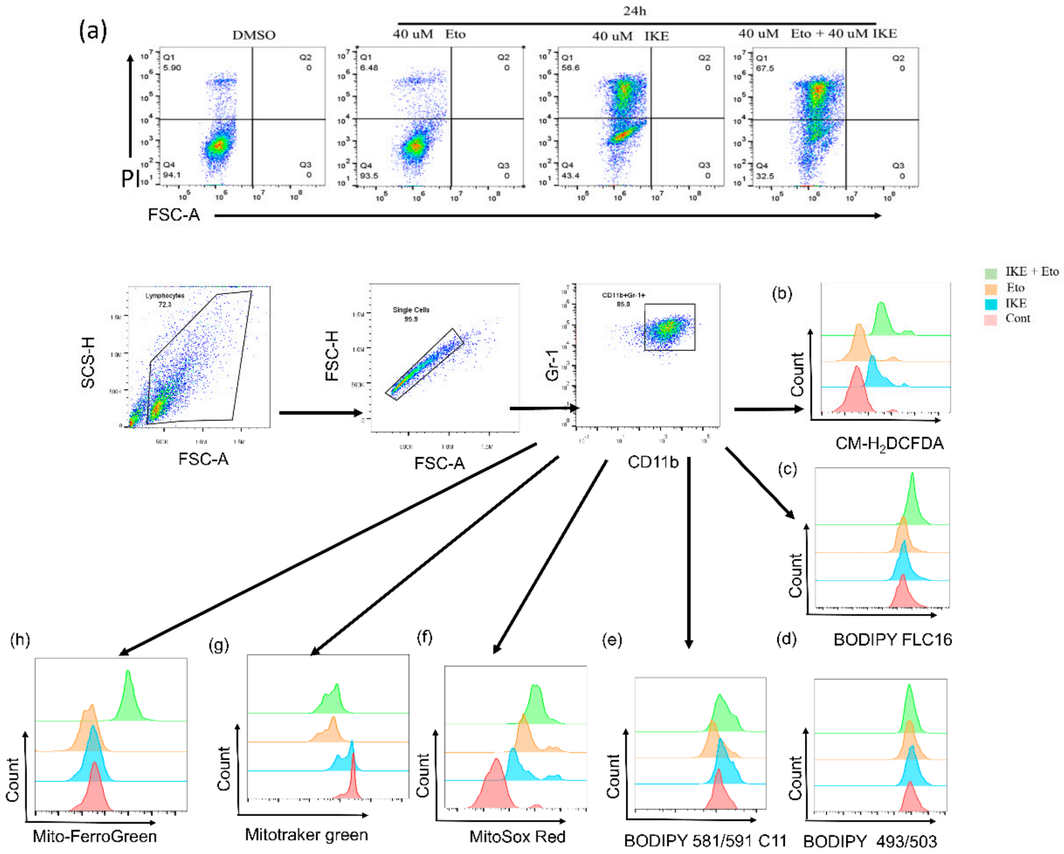

Figure S1. Representative flow cytometry plots illustrating the gating strategy of cell viability ($PI^-FSC\text{-}A^-$) for BM-derived $CD11b^+Gr\text{-}1^+$ MDSCs treated with IKE, or Eto, or both together in vitro for 24 hours (a). Representative flow cytometry plots illustrating the gating strategy of mean fluorescence intensity (MFI) of ROS production (b), fatty acid uptake (c), lipid droplets (d), lipid peroxidation (e), mitochondria superoxide anions (f), mitochondrial mass (g), and mitochondria Fe^{2+} (h) of in vitro BM-derived $CD11b^+Gr\text{-}1^+$ MDSCs.

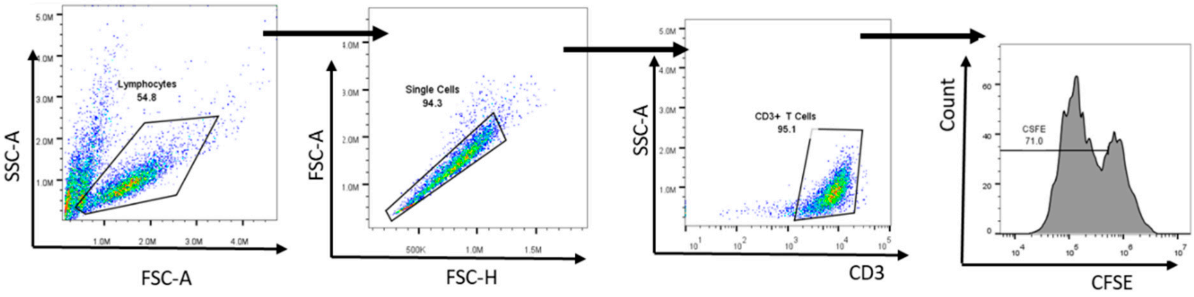

Figure S2. Representative flow cytometry plots illustrating the gating strategy of T cell proliferation in co-cultures of CFSE-labeled $CD3^+$ T cells activated with plate-bound anti-mouse CD3 and anti-mouse CD28 and $CD11b^+Gr\text{-}1^+$ MDSCs at a 2:1 ratio.

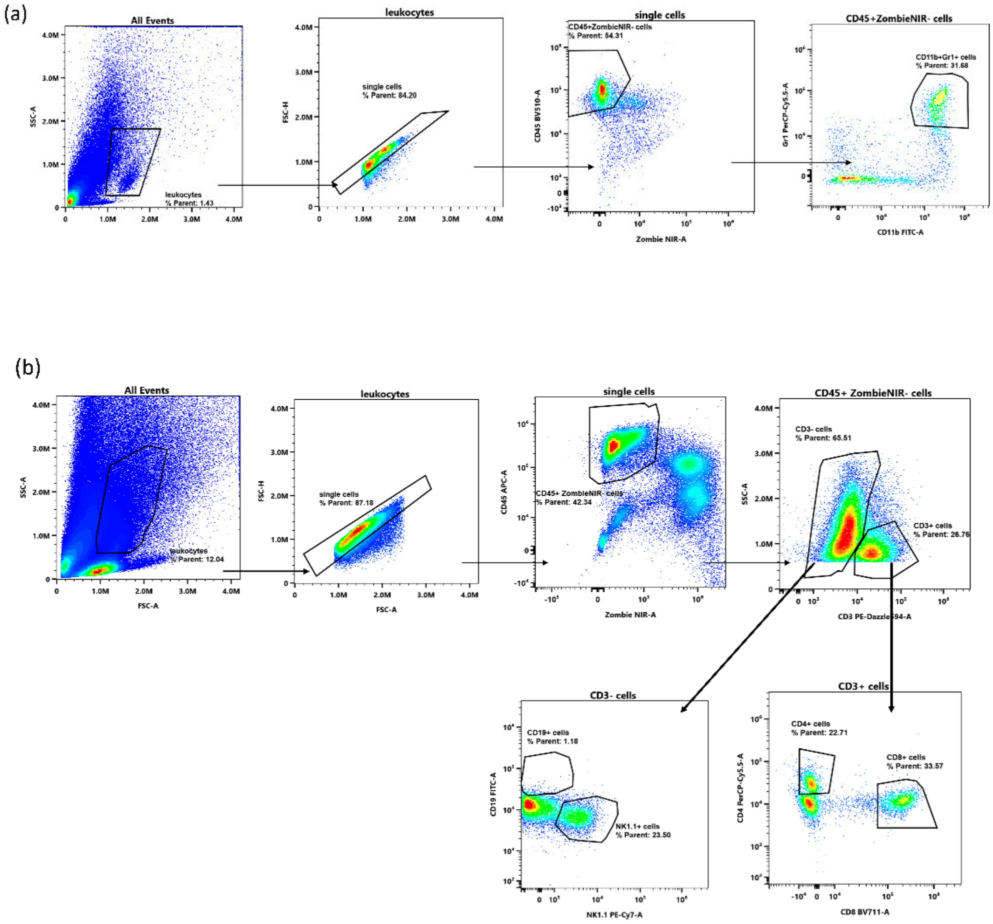

Figure S3. Representative flow cytometry plots illustrating the gating strategy of tumor leukocytes in LLC tumor-bearing mice: CD45+ Leukocytes (CD45$^+$ZombieNIR$^-$Gated on total single cells), MDSCs (CD45$^+$CD11b$^+$Gr-1$^+$), CD3$^+$ T cells (CD45$^+$CD3$^+$), B cells (CD45$^+$CD3$^-$CD19$^+$NK1.1$^-$), NK cells (CD45$^+$CD3$^-$NK1.1$^+$CD19$^-$), CD4$^+$ T cells (CD45$^+$CD3$^+$CD4$^+$CD8$^-$), CD8$^+$ T cells (CD45$^+$CD3$^+$CD4$^-$CD8$^+$).

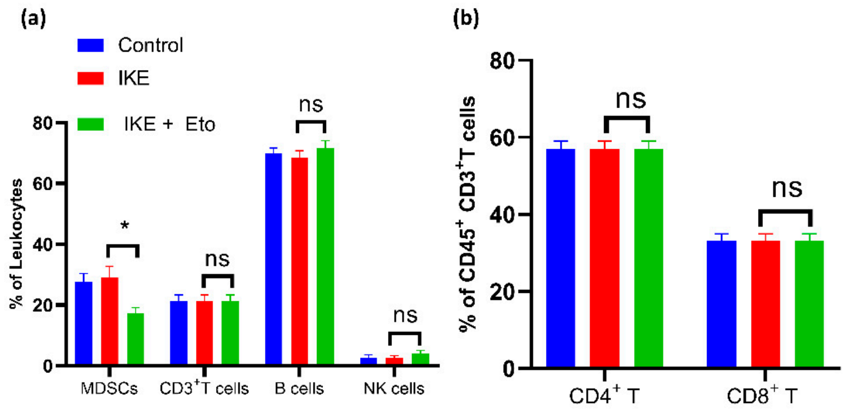

Figure S4. IKE and Eto combined treatment slow tumor growth in LLC-bearing mice. C57BL/6 mice were injected s.c. LLC tumor cells and then daily treated with 10 mg/kg IKE and 15 mg/kg Eto or combined therapy from day 0 to day 21 after tumor injection (n = 4 mice/group). (a) The proportions of splenic immune cells in LLC tumor-bearing mice: MDSCs, $CD3^+$ T cells, B cells, and NK cells. (b) $CD4^+$ T cells, and $CD8^+$ T cells were detected by flow cytometry. Results from three independent experiments (a-b). Statistical analysis was performed using one-way ANOVA. Data are expressed as means±SEM. *$p < 0.05$, ns, no significant difference.

CPT1A- In vitro- Fig 3b

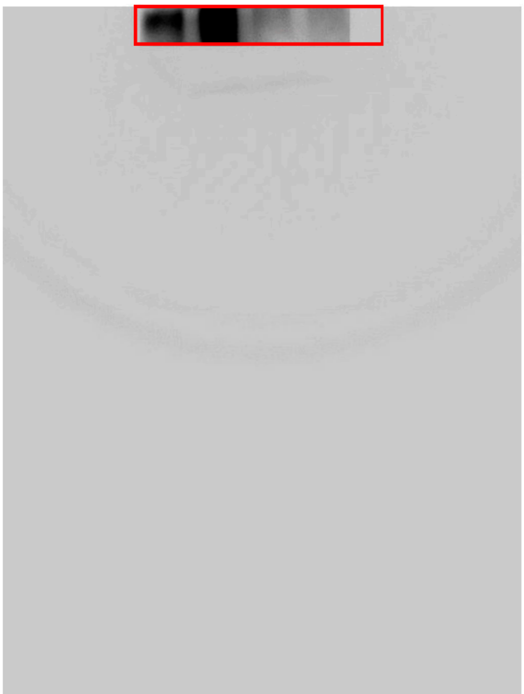

CPT1A- In vivo- Fig 3e

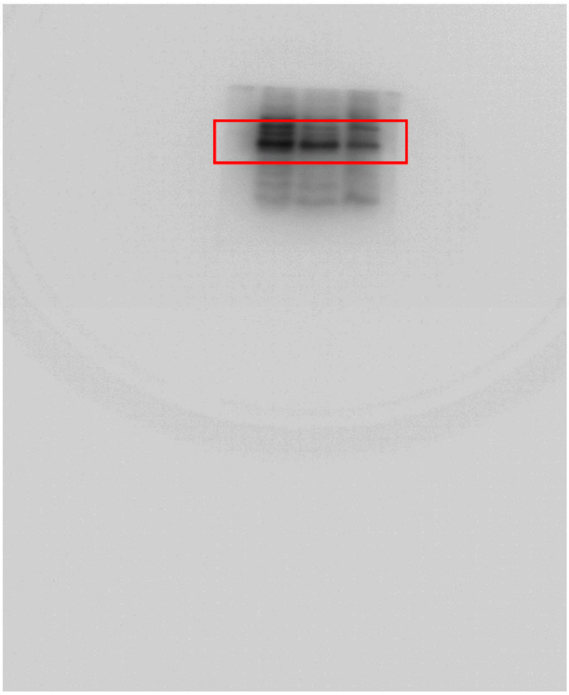

ARG-1- In vitro- Fig 3b

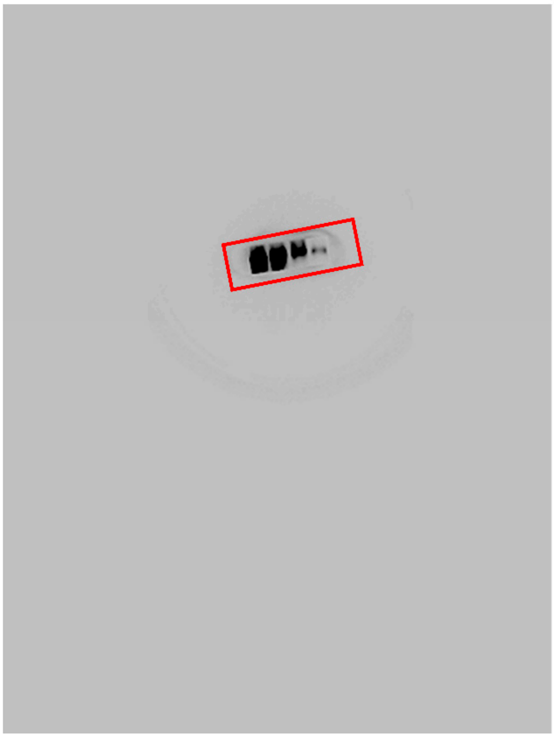

AEG-1- In vivo- Fig 3e

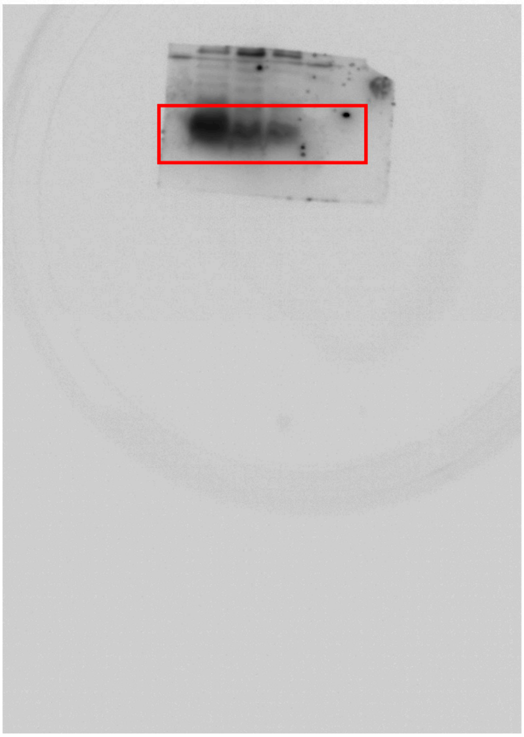

GPX4 In vitro- Fig 3b

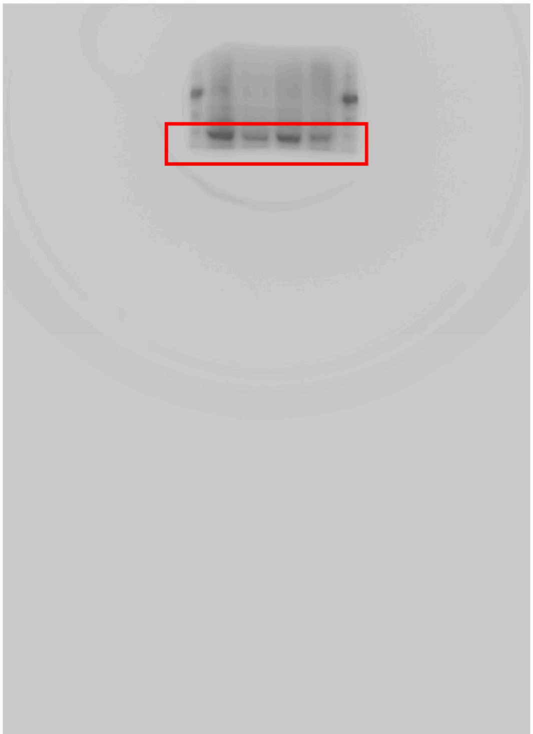

GPX4- In vivo- Fig 3e

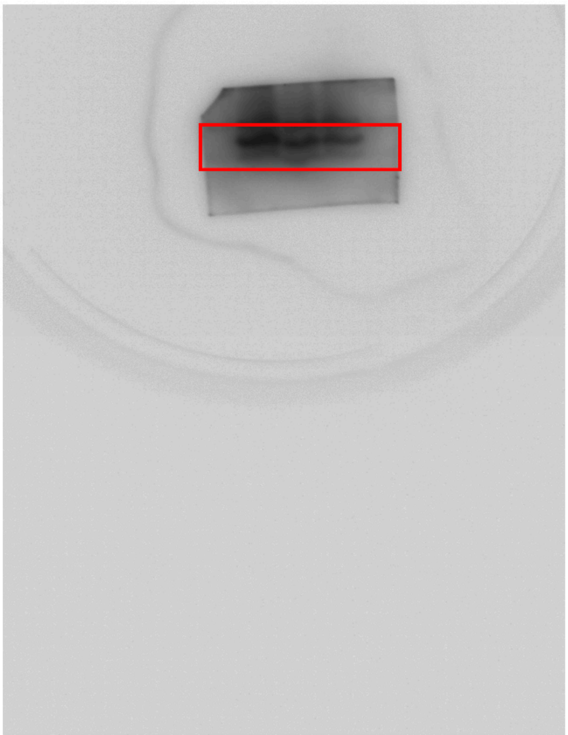

SLC7A11- In vitro- Fig 3b

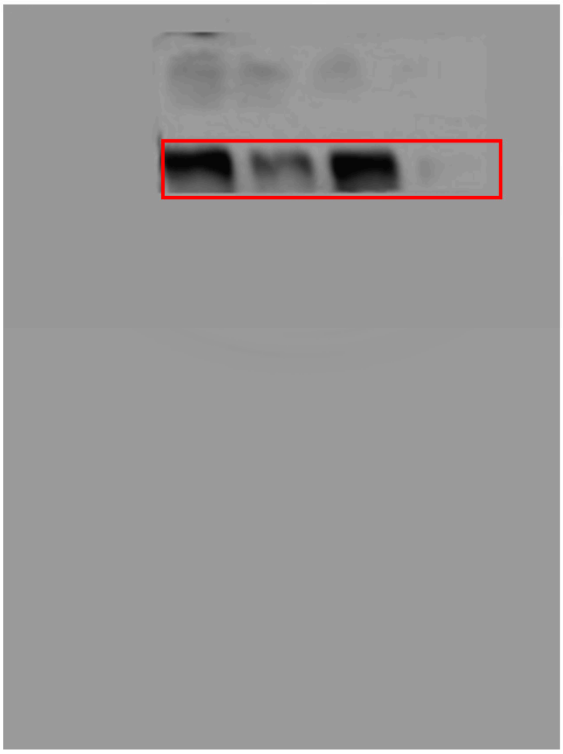

SLC7A11- In vivo- Fig 3c

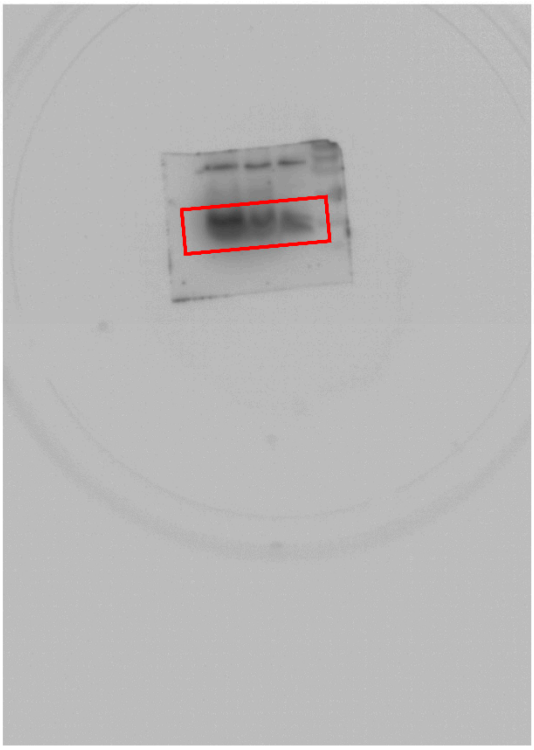

VINCULIN- In vitro- Fig 3b

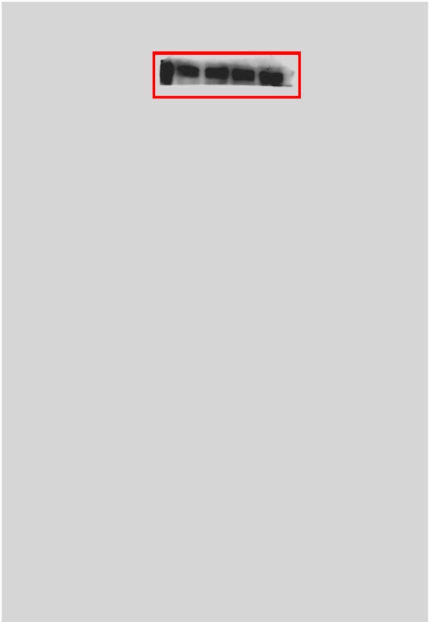

VINCULIN- In vivo- Fig 3e

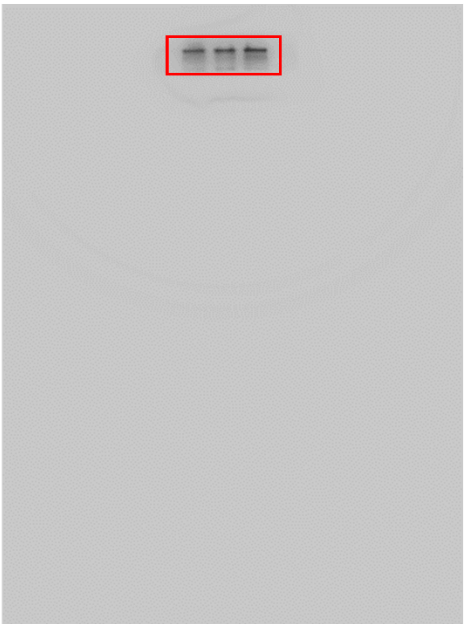

WB: Every subfigure's original complete Western blot figures. After cutting the polyvinylidene difluoride (PVDF) membrane close to each protein's unique size and incubating it with primary and secondary antibodies, we were able to identify the PVDF membrane for each protein independently. Next, we detected the membrane using the Amersham Imager 600 machine. (a) The supplementary western blot data for figure 3 (b). (b) The supplementary western blot data for figure 3 (e).
